# Diagnostic accuracy and severity prediction of monocyte distribution width in sepsis: a retrospective study from the United Arab Emirates

**DOI:** 10.3389/fmed.2026.1891671

**Published:** 2026-08-05

**Authors:** Uzma Sabahat, Liza Thomas, Eram Anwar, Adnan Agha

**Affiliations:** 1 Department of Internal Medicine, Rashid Hospital, Dubai Health, Dubai, United Arab Emirates; 2 College of Medicine and Health Sciences, United Arab Emirates University, Al Ain, United Arab Emirates

**Keywords:** biomarker, monocyte distribution width, procalcitonin, qSOFA, sepsis, United Arab Emirates

## Abstract

**Introduction:**

Monocyte distribution width (MDW) is an early sepsis biomarker derived from the routine complete blood count. Existing evidence is overwhelmingly from European, North American, and East Asian cohorts, with no published Middle Eastern data. We evaluated the diagnostic value of MDW for sepsis identification in a United Arab Emirates (UAE) tertiary care population, comparing it with procalcitonin (PCT), C-reactive protein (CRP), and quick Sequential Organ Failure Assessment (qSOFA).

**Methods:**

We retrospectively reviewed 140 adults admitted to Rashid Hospital, Dubai, with confirmed infections in 2024, classified by Sepsis-3 criteria as sepsis (*n* = 53), septic shock (*n* = 21), or other infections (*n* = 66). MDW was measured on a UniCel DxH 900 analyzer. Receiver operating characteristic curves were compared for MDW, CRP, PCT, WBC, and the MDW + qSOFA and CRP + qSOFA composites; logistic regression identified independent predictors of sepsis.

**Results:**

Median MDW differed across groups: 27.6 (IQR 22.6–34.6) in sepsis, 28.2 (25.4–33.0) in septic shock, and 24.0 (19.9–27.6) in other infections (*p* = 0.001). MDW’s AUC was 0.678 (95% CI 0.589–0.764), behind PCT (0.778) and ahead of CRP (0.652) and WBC (0.590). MDW + qSOFA achieved the highest AUC (0.763, 95% CI 0.683–0.840), indistinguishable from CRP + qSOFA (*p* = 0.550). At the FDA-cleared cutoff ≥ 20, MDW had 90.5% sensitivity and 25.8% specificity, supporting a rule-out role. After adjustment for CRP, PCT, age, and sex, only MDW (aOR 1.08; 95% CI 1.00–1.15; *p* = 0.037) and qSOFA (aOR 2.30; 95% CI 1.32–4.00; *p* = 0.003) remained independent predictors. MDW was also higher in blood culture-positive patients (median 29.5 vs. 25.0; *p* = 0.028), though this attenuated to borderline significance (*p* = 0.051) after excluding the 12 patients without cultures.

**Discussion:**

In this first UAE cohort, MDW behaved consistently with international evidence. Standalone discrimination was moderate, but MDW + qSOFA matched a CRP-based composite at no additional sample/reagent cost. As the comparator group comprised only infected patients, these AUCs reflect an infection-enriched design and are expected to be lower than in unselected emergency-department populations. Region-specific cutoffs and prospective multicenter Gulf evaluations are required for further evaluation.

## Introduction

Sepsis carries an enormous global burden, with an estimated 48.9 million cases and 11 million deaths annually, accounting for almost one in five deaths worldwide (1). The current consensus operationalization, Sepsis-3, defines it as life-threatening organ dysfunction caused by a dysregulated host response to infection, indexed by an increase of two or more points in the Sequential Organ Failure Assessment score (2). Outcomes depend critically on how quickly clinicians recognize it and start antimicrobial therapy, with every hour of delay translating into excess mortality (3).

The current biomarker toolkit has well-established limitations. Although universally available, C-reactive protein levels rise late (peak at 24–48 h) and lack specificity (4, 5). Procalcitonin is more discriminating for bacterial infection but underperforms in immunocompromised hosts and increases in non-infectious inflammatory states, such as post-surgery (6). Both require dedicated immunoassays with turnaround times of 1–2 h (7), which can be the difference between timely antibiotic delivery and a missed therapeutic window.

Monocyte distribution width (MDW) is a more recent diagnostic tool. It quantifies the volumetric dispersion of circulating monocytes and is generated automatically on Beckman Coulter DxH-series analyzers as part of the routine CBC, with no additional draw, reagent, or order needed (8, 9). The biological signal is rapid innate-immune activation: on encountering pathogen-associated molecular patterns, monocytes increase in size, form filopodia, and shift towards CD16-expressing inflammatory phenotypes, producing a heterogeneous population that registers as widened volume distribution within hours of pathogen exposure, well before CRP or PCT can rise (7, 10, 11).

Since the pivotal Crouser report in 2017 (8), the MDW has accumulated a sizeable evidence base. A multicenter European prospective study by Hausfater et al. reported AUCs of 0.81–0.82 in 1,517 ED patients, climbing to 0.86 when paired with WBC (12). Primary studies have produced AUCs spanning roughly 0.52 to 0.96, with most of the variance attributable to spectrum-of-disease and reference-standard choices (13–15). Six systematic reviews and meta-analyses have confirmed its diagnostic reliability, with a pooled sensitivity of 84% (95% CI 79–88%) and SROC AUC of 0.85 reported by Huang et al. (16–18). A consistent thread across studies is the high negative predictive value (often 93–98%), which positions MDW primarily as a rule-out tool (8, 9, 13, 18).

Practical considerations also favor the MDW. The median turnaround time in a cancer-enriched ED cohort was 59 min for MDW, 105 min for CRP, and 111 min for PCT (7). Modelled annual savings at the hospital level from an earlier sepsis identification approach was US$713,000, (19) and real-world implementation in a Paris ED demonstrated meaningful reductions in time-to-antimicrobial (20).

Despite this momentum, the literature has a striking geographical blindspot. In the most comprehensive infection-wide meta-analysis to date, the 28 included studies came only from Europe (*n* = 12), Asia (*n* = 11), and the United States (*n* = 5); to our knowledge, none has been conducted in the UAE (18). Gulf populations differ from those previously studied along several dimensions that plausibly affect MDW performance, including a high background prevalence of diabetes, distinct endemic infectious disease profiles, and demographic heterogeneity from large expatriate communities (17, 21). Optimal MDW thresholds may require regional recalibration, as performance has been shown to vary with both the sepsis criterion applied and the anticoagulant tube used (18).

Against this background, we set out to evaluate the diagnostic accuracy of MDW for sepsis detection and severity prediction in adults at a tertiary-care hospital in the UAE and to compare its performance with that of CRP, PCT, and qSOFA. To our knowledge, this is the first evaluation of this kind in the UAE.

## Methods

### Study design and setting

This was a retrospective, single-center observational study conducted at Rashid Hospital, a 730-bed tertiary care facility in Dubai. The study was approved by the Dubai Scientific Research Ethics Committee (DSREC), Dubai Health Authority (MBRU IRB-2024-675; approval reference DSREC-03/2025_06; approved 14 March 2025), with a formal waiver of individual informed consent granted in view of the retrospective design and the use of de-identified records.

### Population and case ascertainment

We screened consecutive adult patients aged 16 years and older, with no upper age limit, who were admitted to the adult medical wards or the adult intensive care unit between 1 January and 31 December 2024 and who carried an ICD-10 code of R65 (symptoms and signs specifically associated with systemic inflammation and infection) or R65.21 (septic shock) at any point during the admission, reflecting clinically suspected sepsis at presentation. One 15-year-old patient was admitted to and managed on the adult medical service rather than pediatrics, in keeping with local admission practice, and was investigated and treated under the same clinical pathway as the rest of the cohort; this patient was retained on that basis. Coding was verified against the treating-clinician diagnosis documented in the electronic medical record. Because MDW is not used in routine clinical practice at our institution, the reference diagnosis was necessarily established without reference to MDW values, providing inherent blinding of the reference standard to the index test. Patients were excluded if their sepsis was diagnosed more than 48 h after admission (nosocomial), if their records were incomplete or inaccessible, if they had been transferred with an established infection or sepsis already in train, or if they were trauma or pregnant patients. The sample size of 140 was determined *a priori* using the variance approximation of Hanley and McNeil for the area under the ROC curve (22). Assuming an anticipated MDW AUC of 0.70, drawn from pooled meta-analytic estimates (16, 17), a null AUC of 0.50, a two-sided *α* of 0.05, and an approximately 1:1 ratio of cases to controls, 140 patients (74 sepsis vs. 66 other-infection) yields a 95% confidence interval half-width of approximately ±0.09 for the primary AUC estimate and ≥ 80% power to reject the null hypothesis that the AUC equals 0.50. This sample size was also judged adequate to support the pre-specified secondary comparisons of MDW with CRP, PCT, WBC, and the combined MDW + qSOFA model, and corresponds to the projected case volume under the internal medicine service over the study period.

### Data and definitions

From each chart, we extracted the following data: demographics, admission date, discharge diagnosis, source of infection, quick SOFA (qSOFA), Systemic Inflammatory Response Syndrome (SIRS) criteria, and admission laboratory values for CRP (mg/L), PCT (ng/mL), white-cell count (×10^3^/μL), and MDW. We also recorded microbiology (blood, urine, wound, and respiratory cultures). Blood culture status was determined from documented growth in the laboratory information system; patients in whom blood culture was not sent (*n* = 12) were classified as culture-negative for the purposes of analysis, since absence of a culture cannot be taken as evidence of bacteremia. MDW was read directly from the UniCel DxH 900 analyzer (Beckman Coulter, Brea, CA, USA) as part of the routine CBC with differential. Samples were collected into K2-EDTA (dipotassium EDTA) tubes in line with the routine hematology collection protocol at our institution. Patients were assigned to one of three diagnostic groups: (i) sepsis, meeting Sepsis-3 criteria with a SOFA increase of ≥ 2 points (2); (ii) septic shock, meeting sepsis criteria with vasopressor requirement and lactate > 2 mmol/L (2); and (iii) other infection, where infection was confirmed but sepsis criteria were not met. Because this was a retrospective study, group assignment was based on the treating team’s Sepsis-3-consistent diagnosis documented in the medical record and corroborated by ICD-10 coding, rather than on an investigator-reconstructed total SOFA score; the treating physicians made these determinations in real time with full access to organ-function data. The qSOFA analyzed as a predictor was derived from its three bedside components (respiratory rate, altered mentation, and systolic blood pressure), which were available for all but one patient. A binary sepsis indicator (sepsis + septic shock vs. others) was created for the primary ROC analysis.

### Statistical analysis

Distributions were assessed using the Shapiro–Wilk test; continuous biomarkers were uniformly non-normal, so non-parametric methods were used throughout. Continuous variables are reported as median (interquartile range, IQR) and categorical variables as n (%). Three-group comparisons used the Kruskal–Wallis test with Dunn’s post-hoc test and Bonferroni adjustment; categorical comparisons used the χ^2^ test, and two-group comparisons used the Mann–Whitney *U* test. Missing laboratory data were minimal (procalcitonin in 3 patients, 2.1%; qSOFA and SIRS in 1 patient each, 0.7%), reflecting tests not ordered by the treating team rather than any systematic pattern; a complete-case approach was used and the denominator is reported alongside each affected analysis. The diagnostic performance of MDW, CRP, PCT, WBC, and the two logistic regression-derived composites MDW + qSOFA and CRP + qSOFA was characterized using ROC analysis. For each model, we reported the AUC with 95% CI, Youden-optimal cutoff, sensitivity, specificity, positive and negative predictive values, and positive and negative likelihood ratios. Pairwise AUC comparisons used the non-parametric method of DeLong et al. (23). Predictors for the multivariable models were pre-specified on clinical grounds rather than selected by data-driven stepwise procedures; with 74 sepsis events and six candidate predictors in the full model, the events-per-variable ratio was 12.3, exceeding the conventional minimum of 10. Model optimism was quantified using Harrell’s bootstrap procedure (2,000 resamples), reporting the apparent AUC, mean optimism, and optimism-corrected AUC; calibration was assessed using the Hosmer–Lemeshow goodness-of-fit test and the bootstrap-estimated calibration slope. Univariate and multivariate logistic regression analyses were used to identify independent predictors of sepsis, reporting odds ratios (OR) with 95% confidence intervals (CI). Multicollinearity was assessed using variance inflation factors (VIF). Inter-biomarker associations were quantified using Spearman’s p. Analyses were performed using IBM SPSS Statistics version 31 (IBM Corp., Armonk, NY), with bootstrap internal validation performed in Python 3.11. A two-sided *p* < 0.05 was considered statistically significant.

## Results

### Patient characteristics

**Figure 1:**
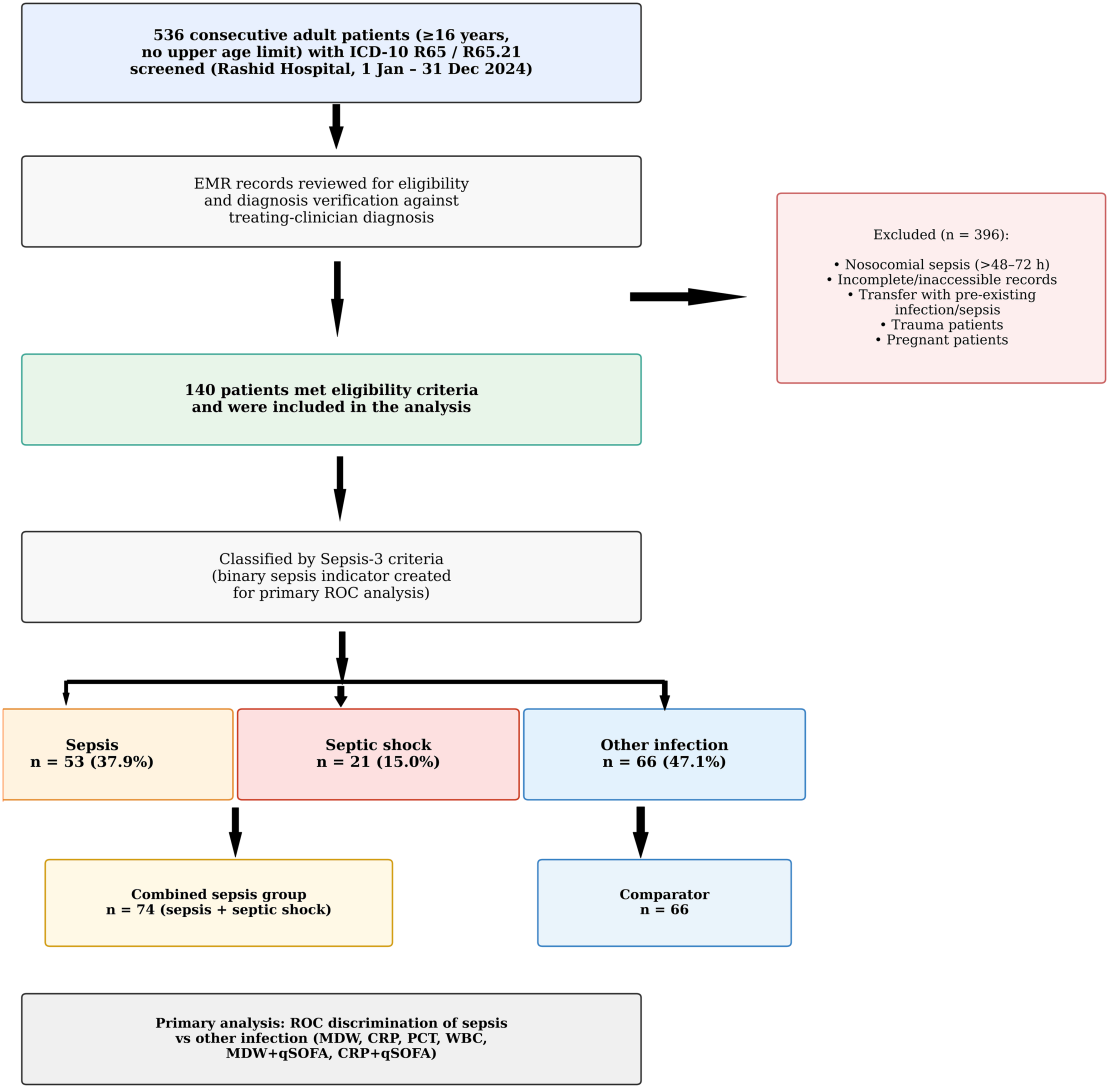
Patient flow diagram. Of 536 consecutive admissions with an ICD-10 R65/R65.21 code screened during 2024, 396 were excluded (nosocomial sepsis diagnosed > 48–72 h after admission; incomplete or inaccessible records; transfer with pre-existing infection or sepsis; trauma; or pregnancy), leaving 140 patients for analysis. Patients were classified by Sepsis-3 criteria into sepsis (*n* = 53), septic shock (*n* = 21), and other infection (*n* = 66); the combined sepsis group (*n* = 74) was compared with the other-infection group (*n* = 66) in the primary ROC analysis.

Of 536 admissions screened, 140 patients met the eligibility criteria and were included; the flow of participants is summarized in Figure 1. The cohort comprised 53 (37.9%) with sepsis, 21 (15.0%) with septic shock, and 66 (47.1%) with other confirmed infections. The mean age was 57.8 ± 21.9 years (range, 15–94). The cohort comprised 80 men (57.1%) and 60 women (42.9%). The septic shock group was the oldest (median 73 years, IQR 61–81), and the other-infections group was the youngest (median 58.5 years, IQR 36–71; *p* = 0.031), but sex distribution was similar across groups (*p* = 0.879). Respiratory infection (lungs/pneumonia) was the most common source (54 patients, 38.6%), followed by urinary tract infection (18, 12.9%) and gastrointestinal tract infection (13, 9.3%). The baseline characteristics of the groups are shown in Table 1; an extended version, including SIRS scores and the full source-of-infection breakdown, is provided in Supplementary Table S1.

**Table 1:** Baseline characteristics stratified by diagnosis group.

Variable	Sepsis (*n* = 53)	Septic shock (*n* = 21)	Other (*n* = 66)	*p*
Age, years, median (IQR)	61 (38–79)	73 (61–81)	58.5 (36–71)	0.031
Male sex, *n* (%)	29 (54.7)	12 (57.1)	39 (59.1)	0.879
MDW, median (IQR)	27.6 (22.6–34.6)	28.2 (25.4–33.0)	24.0 (19.9–27.6)	0.001
CRP, mg/L, median (IQR)	210 (110–315)	166 (113–263)	120 (61–208)	0.007
PCT, ng/mL, median (IQR)	2.1 (0.5–11.6)	14.4 (2.8–50.6)	0.3 (0.1–1.6)	<0.001
WBC, ×10^3^/μL, median (IQR)	12.4 (10.1–17.2)	15.0 (8.9–26.9)	12.1 (8.0–16.0)	0.168
qSOFA, median (IQR)	1 (0–1)	2 (1–2)	0 (0–1)	<0.001
Blood culture positive, n (%)	16 (30.2)	7 (33.3)	6 (9.1)	0.006

Continuous variables are median (IQR); categorical variables are n (%). p-values are from Kruskal–Wallis (continuous) or χ^2^ (categorical). MDW, monocyte distribution width; CRP, C-reactive protein; PCT, procalcitonin; WBC, white blood cell count; qSOFA, quick Sequential Organ Failure Assessment. Sources of infection and SIRS scores are tabulated in Supplementary Table S1.

### Biomarker levels across severity groups

**Figure 2:**
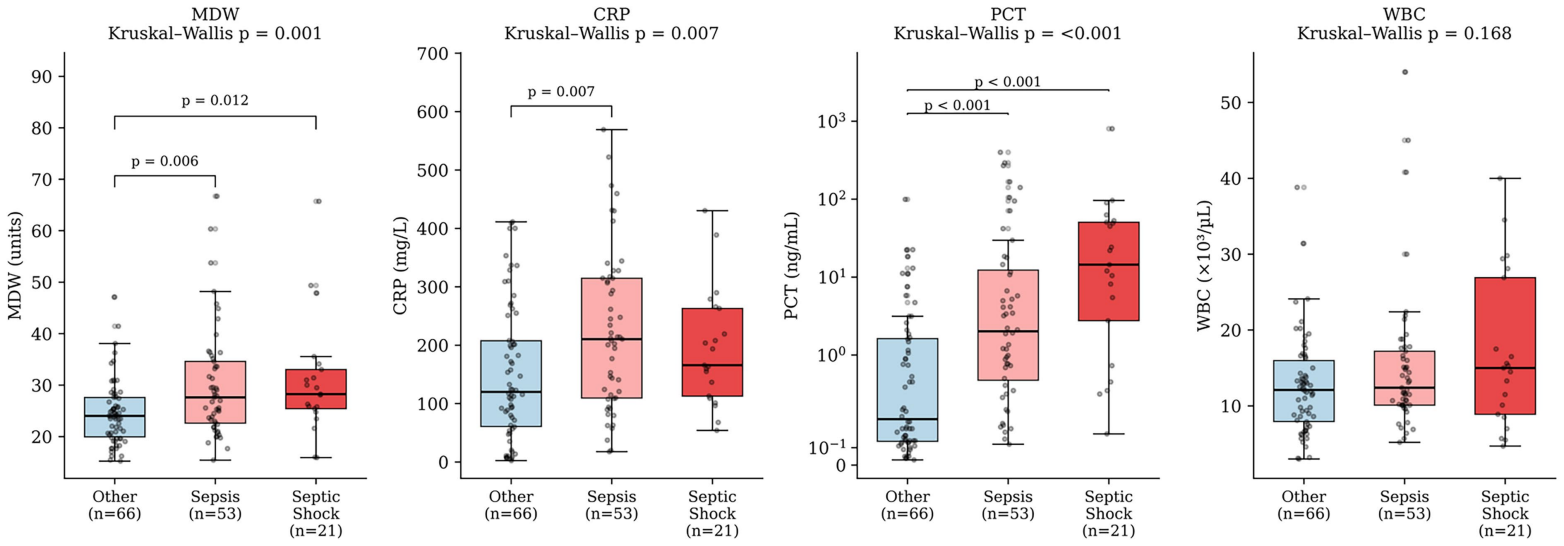
Distribution of biomarker values across the three diagnosis groups. Box-and-whisker plots show MDW, C-reactive protein (CRP), procalcitonin (PCT) and white blood cell count (WBC) for the other-infection (*n* = 66), sepsis (*n* = 53) and septic shock (*n* = 21) groups. Boxes show median and IQR; whiskers extend to 1.5 × IQR; individual data points are overlaid. *p*-values above brackets are from Dunn’s post-hoc test with Bonferroni adjustment following a significant overall Kruskal–Wallis test. MDW, CRP and PCT differed significantly across groups; WBC did not.

Kruskal–Wallis tests demonstrated significant between-group differences for MDW (*H* = 13.50, *p* = 0.001), CRP (*H* = 9.88, *p* = 0.007) and PCT (*H* = 34.62, *p* < 0.001), but not for WBC (*H* = 3.56, *p* = 0.168). Dunn’s *post-hoc* testing with Bonferroni adjustment localized the MDW difference: both sepsis (*p* = 0.006) and septic shock (*p* = 0.012) had higher MDW than the other-infection group, but sepsis and septic shock did not differ (*p* = 1.000). The CRP signal was driven only by sepsis (*p* = 0.007). PCT showed the strongest separation, with both sepsis (*p* < 0.001) and septic shock (*p* < 0.001) markedly elevated compared to the other-infection group. WBC showed no significant pairwise differences (Figure 2).

### Diagnostic performance of MDW for sepsis

**Figure 3:**
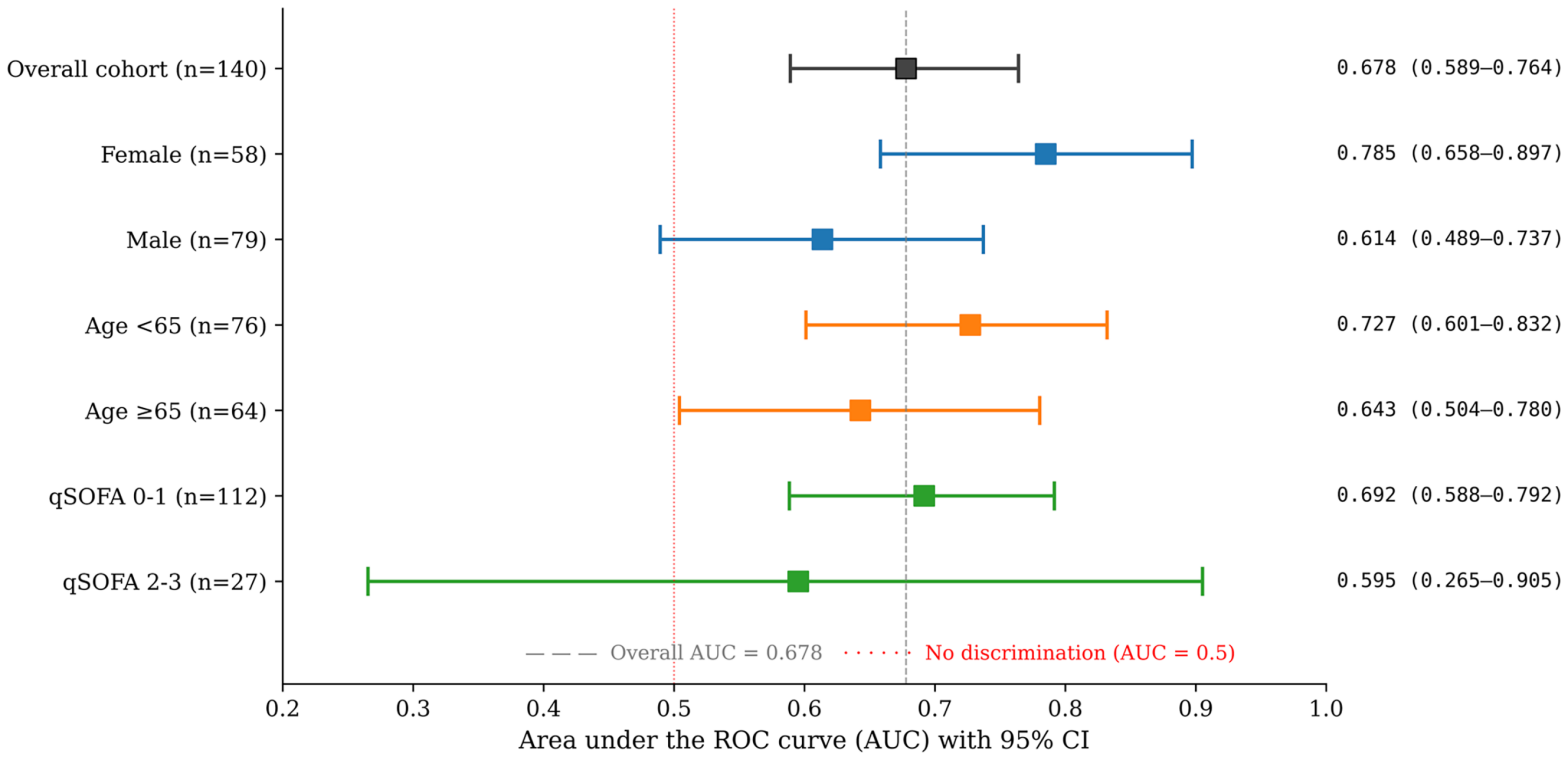
Subgroup analysis of MDW diagnostic performance. Forest plot of MDW area under the ROC curve (AUC) with 95% confidence intervals across age, sex and qSOFA subgroups. MDW performed numerically best in female patients (AUC 0.785; 95% CI 0.658-0.897) and patients aged < 65 years (AUC 0.727; 95% CI 0.601-0.832). The qSOFA 2-3 subgroup estimate (*n* = 27) is unstable owing to small sample size and group imbalance, and should be interpreted with caution. Dashed grey line denotes the overall cohort AUC of 0.678; dotted red line denotes no discrimination (AUC = 0.5).

For discrimination of the combined sepsis group (sepsis + septic shock, *n* = 74) from other infections (*n* = 66), MDW achieved an AUC of 0.678 (95% CI 0.589–0.764), compared with PCT 0.778 (0.701–0.848), CRP 0.652 (0.554–0.741), and WBC 0.590 (0.496–0.680) (Table 2 and Figure 3). DeLong’s test confirmed that PCT outperformed MDW (Z = 2.18, *p* = 0.029), whereas MDW and CRP were statistically indistinguishable (*Z* = 0.91, *p* = 0.364).

**Table 2:** Diagnostic performance of biomarkers and combined models for sepsis detection.

Model	AUC (95% CI)	Cutoff	Sens	Spec	PPV	NPV	+LR	–LR
MDW	0.678 (0.589–0.764)	25.43	63.5	66.7	68.1	62.0	1.91	0.55
CRP	0.652 (0.554–0.741)	136.5	68.9	56.1	63.8	61.7	1.57	0.55
PCT*	0.778 (0.701–0.848)	0.39	86.5	56.3	69.6	78.3	1.98	0.24
WBC	0.590 (0.496–0.680)	14.4	46.0	72.7	65.4	54.6	1.68	0.74
MDW + qSOFA*	0.763 (0.683–0.840)	0.44	76.7	65.2	70.9	71.7	2.20	0.36
CRP + qSOFA*	0.743 (0.659–0.821)	0.43	80.8	56.1	67.1	72.6	1.84	0.34
MDW ≥ 20 (fixed)	0.678†	20.0	90.5	25.8	57.8	70.8	1.22	0.37
MDW ≥ 20.5 (fixed)	0.678†	20.5	87.8	27.3	57.5	66.7	1.21	0.45

*N = 138 for PCT and *N* = 139 for combined models, owing to missing values. †Same ROC curve as MDW; only the cutoff is varied. Sens, sensitivity (%); Spec, specificity (%); PPV/NPV, positive/negative predictive value (%); +LR/−LR, positive/negative likelihood ratio.

The Youden-optimal MDW cutoff in this cohort was 25.43, yielding 63.5% sensitivity, 66.7% specificity, 68.1% PPV, 62.0% NPV, and +LR 1.91. At the FDA-cleared cutoff of ≥ 20, sensitivity rose to 90.5%, but specificity fell to 25.8%, with an NPV of 70.8%. The slightly higher fixed cutoff of ≥ 20.5 provided a sensitivity of 87.8% and specificity of 27.3%.

The combined MDW + qSOFA logistic regression score produced the highest discrimination overall (AUC 0.763; 95% CI 0.683–0.840) and the best positive likelihood ratio (+LR 2.20). However, it was not significantly different from the CRP + qSOFA (AUC 0.743; DeLong *p* = 0.550).

### Inter-biomarker correlations

Spearman analysis revealed moderate positive associations between MDW and PCT (*ρ* = 0.621, *p* < 0.001) and CRP (ρ = 0.407, *p* < 0.001). MDW correlated only weakly and non-significantly with the clinical severity scores of qSOFA (ρ = 0.074, *p* = 0.39) and SIRS (ρ = 0.008, *p* = 0.93). In contrast, qSOFA and SIRS were moderately correlated with each other (ρ = 0.558, *p* < 0.001), and WBC correlated with SIRS (ρ = 0.389, *p* < 0.001). Taken together, this pattern suggests that MDW carries inflammatory information that complements but is pathophysiologically distinct from the bedside severity scores (Supplementary Figure S1).

### Logistic regression

Every biomarker we tested was a significant univariate predictor of sepsis-group membership: MDW (OR 1.09; 95% CI 1.04–1.15; *p* < 0.001), CRP (OR 1.004; 95% CI 1.001–1.007; *p* = 0.004), PCT (OR 1.04; 95% CI 1.01–1.07; *p* = 0.012), WBC (OR 1.05; 95% CI 1.00–1.10; *p* = 0.033) and qSOFA (OR 2.43; 95% CI 1.52–3.89; *p* < 0.001).

After adjusting for qSOFA, age, and sex, MDW retained independent significance (adjusted OR 1.11; 95% CI 1.05–1.18; *p* < 0.001; AIC 163.7). In the full model containing MDW, CRP, PCT, qSOFA, age, and sex, only MDW (adjusted OR 1.08; 95% CI 1.00–1.15; *p* = 0.037) and qSOFA (adjusted OR 2.30; 95% CI 1.32–4.00; *p* = 0.003) remained independent predictors, while CRP and PCT lost significance (*p* = 0.265 and *p* = 0.116, respectively). No collinearity was observed (all VIFs < 1.8; Table 3).

**Table 3:** Logistic regression models predicting sepsis group membership.

Variable	Univariate OR (95% CI)	*p*	Full model adjusted OR (95% CI)	*p*
MDW (per unit)	1.09 (1.04–1.15)	<0.001	1.08 (1.00–1.15)	0.037
CRP (per mg/L)	1.004 (1.001–1.007)	0.004	1.002 (0.998–1.006)	0.265
PCT (per ng/mL)	1.04 (1.01–1.07)	0.012	1.024 (0.994–1.055)	0.116
WBC (per ×10^3^/μL)	1.05 (1.00–1.10)	0.033	—	—
qSOFA (per point)	2.43 (1.52–3.89)	<0.001	2.30 (1.32–4.00)	0.003
Age (per year)	—	—	1.015 (0.996–1.035)	0.119
Male sex	—	—	0.84 (0.36–1.98)	0.698

Full model AIC: 157.4; all VIF < 1.8. Reduced model (MDW + qSOFA + Age + Sex) AIC: 163.7. OR, odds ratio; CI, confidence interval.

Internal validation confirmed limited overfitting. For the full model, the apparent AUC of 0.807 reduced to an optimism-corrected AUC of 0.772 (mean optimism 0.035), with a bootstrap calibration slope of 0.96 and a non-significant Hosmer–Lemeshow statistic (χ^2^ = 10.28, df = 8, *p* = 0.25), indicating good calibration (Supplementary Figure S2). The reduced model (MDW + qSOFA + age + sex) showed an apparent AUC of 0.770 and an optimism-corrected AUC of 0.746, and the MDW + qSOFA composite was the most stable, with minimal optimism (apparent AUC 0.764, optimism-corrected 0.757). The fitted composite in this cohort was logit(P) = −3.067 + 0.0895 × MDW + 0.9096 × qSOFA; we provide these coefficients for reproducibility and external benchmarking, but emphasize that they were derived in a single-center cohort and require external validation before any clinical deployment.

### Blood culture positivity and subgroup analyses

Of the 140 patients, 29 (20.7%) had positive blood cultures. MDW was significantly higher in culture-positive than in culture-negative patients (median 29.5 vs. 25.0; Mann–Whitney *U* = 2,037; *p* = 0.028). Positivity rates were comparable in sepsis (30.2%) and septic shock (33.3%) but substantially lower in the other-infection group (9.1%; χ^2^ = 10.36, *p* = 0.006). In a sensitivity analysis excluding the 12 patients without blood cultures, the association was directionally unchanged but attenuated to borderline significance (median 29.5 vs. 25.3; *U* = 1,779; *p* = 0.051; *n* = 128; Supplementary Table S2), indicating that this finding is sensitive to the handling of patients without cultures and should be interpreted with appropriate caution.

**Figure 4:**
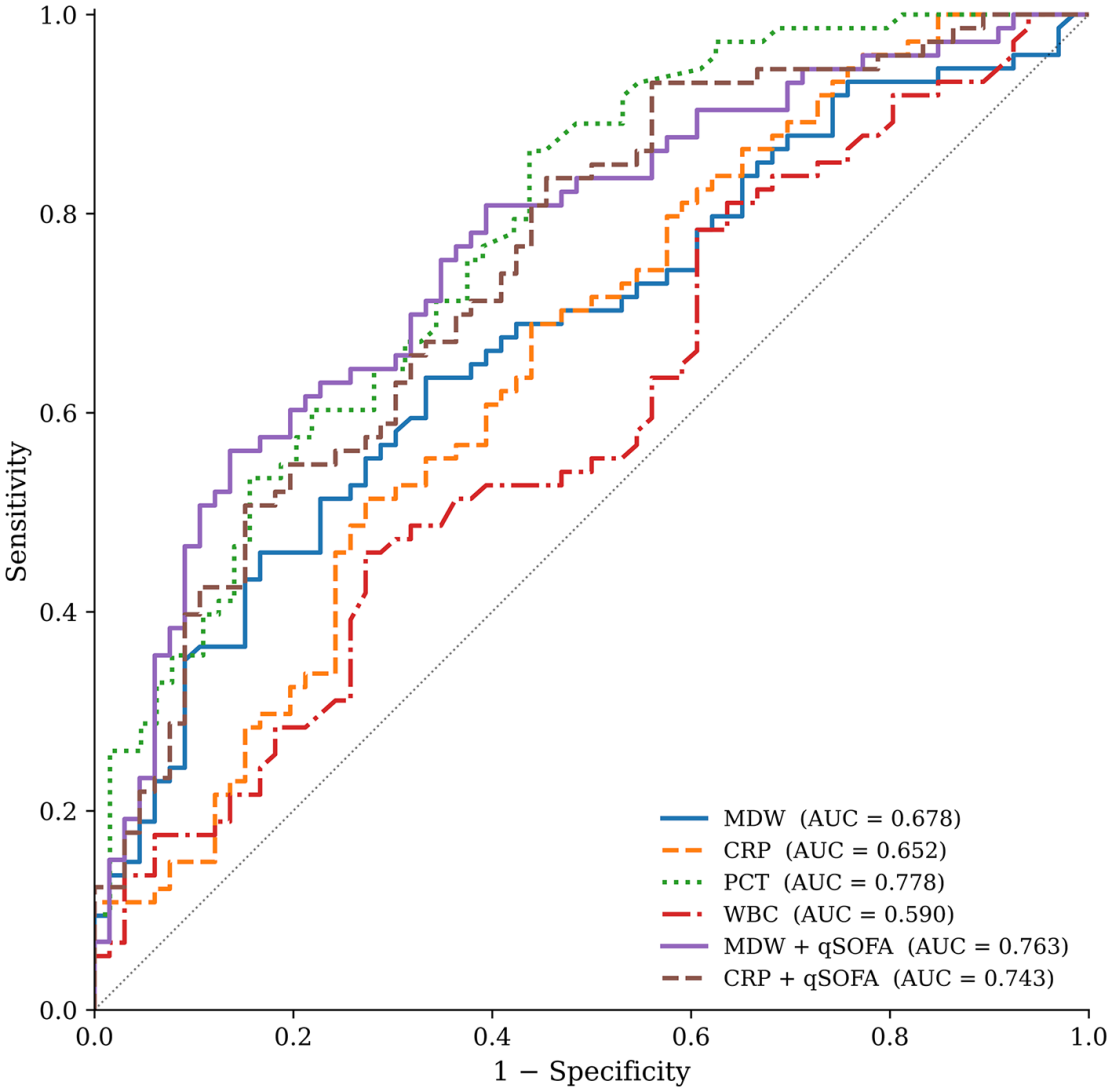
Receiver operating characteristic (ROC) curves for sepsis detection. Curves show the diagnostic performance of MDW, CRP, PCT, WBC, and the combined logistic-regression models MDW + qSOFA and CRP + qSOFA for discriminating sepsis (sepsis + septic shock; *n* = 74) from other infections (*n* = 66). The combined MDW + qSOFA model achieved the highest AUC (0.763; 95% CI 0.683-0.840); PCT alone achieved AUC 0.778 (0.701-0.848). DeLong’s test demonstrated PCT outperformed MDW (*p* = 0.029), with no significant difference between MDW + qSOFA and CRP + qSOFA (*p* = 0.550). qSOFA, quick Sequential Organ Failure Assessment.

Prespecified subgroup analyses suggested heterogeneity in MDW performance. Discrimination was numerically higher in women (AUC 0.785; 95% CI 0.658–0.897) than in men (0.614; 0.489–0.737), and in patients aged < 65 years (0.727; 0.601–0.832) than in those aged ≥ 65 years (0.643; 0.504–0.780). In patients with qSOFA 0–1 (*n* = 112), the AUC was 0.692 (0.588–0.792), while the small qSOFA 2–3 subgroup (*n* = 27) gave an unstable estimate (AUC 0.595; 0.265–0.905) (Figure 4). Among the 67 patients with available discharge dates, the median length of stay was 8 days (mean 12.5 ± 14.9), with no meaningful correlation between MDW and length of stay (Spearman *ρ* = −0.138; *p* = 0.272).

## Discussion

To our knowledge, this is the first study to evaluate MDW as a sepsis diagnostic and severity biomarker in a UAE population. The headline findings are straightforward: MDW is significantly elevated in sepsis and septic shock compared with other infections, retains independent predictive value when adjusted alongside qSOFA, and is in accordance with blood culture positivity. Together, these results begin to fill the geographical gap noted in recent comprehensive reviews, which identified a near-complete absence of MDW validation data from Middle Eastern populations (17, 21).

Our MDW AUC of 0.678 sits at the lower end of the published range (0.52 to 0.96 across ED and ICU studies) (10). That is not surprising. Spectrum bias is well documented in diagnostic biomarker studies; when the comparison group is already enriched for infection or suspected sepsis, the discriminative range narrows because the very low MDW values seen in uninfected controls are simply absent. Cusinato et al. reported a similarly modest AUC of 0.59 in 2,570 patients already on a London sepsis pathway (24), and Kim et al. found an AUC of only 0.52 in patients with suspected sepsis (25). In contrast, the highest AUCs (> 0.90) were observed in unselected ED populations, where uninfected controls drove the gradient, as in the 2,215-patient cohort reported by Agnello et al. (AUC 0.964) (14) and the Korean cohort of Park et al. (AUC 0.869) (26).

Our population-specific Youden cutoff of 25.43 was higher than the conventional 20.0–21.5. Again, the infection-enriched baseline likely contributed, and our value is in line with ICU-based studies that have adopted higher thresholds; Piva et al. found 24.63 to be optimal in 506 ICU patients (AUC 0.785) (27), and Polilli et al. recommended dual cutoffs of > 23 for ruling-in and ≤ 20 for ruling-out (28). The sample collection tube type also matters. Malinovska et al. showed that the optimal cutoff varies with the anticoagulant used, clustering around 20.0–20.1 units for K2-EDTA and 21.5 units for K3-EDTA (18). Our samples were collected in K2-EDTA; the Youden-derived cutoff of 25.43 therefore cannot be explained by a K3-EDTA tube effect, and its elevation above the usual K2-EDTA optimum instead reflects the infection-enriched comparator and the regional factors noted below. This cutoff is specific to K2-EDTA and would require recalibration in laboratories using K3-EDTA.

However, at the established cutoff of ≥ 20, MDW showed 90.5% sensitivity in our cohort, in keeping with its principal clinical role as a high-sensitivity rule-out tool. Our value is consistent with the NPVs of 93–97% reported by Crouser et al. at similar cutoffs (8, 9), the 94% sensitivity and 88% specificity reported by Královcová et al. when MDW was combined with organ dysfunction markers (AUC 0.96) (13), and the 91% sensitivity and 98% NPV reported by Choi et al. in a cancer-enriched cohort (7). In a triage workflow, this matters: a low MDW could plausibly steer clinicians away from costly downstream sepsis workup, while a raised value would flag the patient for prompt evaluation.

In our cohort, PCT outperformed MDW as a stand-alone marker (AUC 0.778 vs. 0.678), which is consistent with several head-to-head studies. Woo et al. reported a similar pattern with MDW AUC 0.71, CRP 0.75, and PCT 0.76 (29). Li and colleagues found overlapping accuracy for MDW (AUC 0.82) and PCT (AUC 0.87) in a Taiwanese ED (30). The 2023 meta-analysis by Motawea et al., which compared MDW directly with PCT across five studies, reported summary AUCs of 0.790 and 0.760, respectively, suggesting near equivalence (31). However, PCT requires a dedicated immunoassay, takes longer to return, and incurs incremental costs (7, 11).

Monocyte distribution width is a particularly useful diagnostic marker of sepsis when combined with bedside scoring. In our analysis, MDW + qSOFA gave the highest AUC of any model (0.763) and was statistically indistinguishable from CRP + qSOFA (DeLong *p* = 0.550). This echoes the findings of Woo et al., who found that adding MDW to qSOFA (AUC 0.67) increased the AUC to 0.76 (29); Crouser et al., who showed that MDW extended early sepsis detection beyond either SIRS or qSOFA alone (32); and Lin et al., who reported c-statistics of 0.886 in older adults using a four-component score that included MDW (33). The practical implication is that the freely available MDW can complement dedicated biomarker assays, especially when paired with a structured clinical assessment.

Our multivariate results are internally consistent. After adjustment, MDW and qSOFA were the only variables that retained significance; CRP and PCT did not. The moderate Spearman correlations of MDW with PCT (*ρ* = 0.621) and CRP (ρ = 0.407) suggest substantial overlap in the inflammatory information these markers carry, which is what one would expect when adjustment attenuates their independent contributions. In contrast, the weak correlation of MDW with qSOFA (ρ = 0.074) supports the interpretation that MDW captures something pathophysiologically distinct from hemodynamic and neurological deterioration, specifically monocyte morphological activation (10, 11).

Monocyte distribution width was significantly higher in blood culture-positive patients than in blood culture-negative patients (median 29.5 vs. 25.0; *p* = 0.028). This is in line with Jo et al., who reported that MDW values below 20 were overwhelmingly associated with negative cultures and that culture-positive patients had higher MDW than contamination or negative culture groups (34). Goldner et al. showed that MDW > 28.99 supported a septic over cardiogenic explanation for shock (35).

The graded elevation of median MDW across our severity groups (other infection 24.0, sepsis 27.6, and septic shock 28.2) mirrors the dose–response pattern reported elsewhere. Morales Indiano et al. reported medians of 19.70, 27.20 and 31.10 in non-septic, sepsis and septic shock ICU patients, respectively, (36). Piva et al. found a similar gradient in their ICU cohort (27). The absence of a sepsis-to-septic-shock difference in our cohort hints at a plateau effect at higher severities, as noted by Polilli et al. (28, 37).

Our subgroup analyses found numerically better MDW performance in women (AUC 0.785) and younger patients (< 65 years; AUC 0.727). These results are exploratory: the confidence intervals overlap, subgroup sample sizes are small, and the pattern is not seen across all published cohorts. These subgroup differences are hypothesis-generating only: confidence intervals are wide and overlapping, the subgroups are small, and no formal interaction testing was powered, so they should not guide clinical use. They nonetheless align with the observations of Theodoridis et al. about possible sex- and age-related differences in monocyte response patterns (38, 39), and warrant confirmation in larger cohorts.

## Limitations

This study has several limitations. First, the retrospective, single-center design limits causal inference and external generalizability. Second, the sample size of 140, although adequate for the primary AUC analysis, constrained subgroup analysis. Third, by including only patients with confirmed infection, we eliminated the lower-MDW uninfected comparator group that drives the highest published AUCs, a deliberate trade-off, but one that compresses our discriminative range. Fourth, although samples were collected in K2-EDTA, our Youden-derived cutoff of 25.43 is specific to this anticoagulant and to our infection-enriched cohort, and should not be transferred to laboratories using K3-EDTA without recalibration (18). Fifth, systematically recorded comorbidity and immunological data were not available. Conditions prevalent in Gulf populations; notably diabetes mellitus, chronic kidney disease, malignancy, and immunosuppression; together with prior antibiotic exposure and corticosteroid therapy, are known to influence monocyte morphology and inflammatory biomarker behavior; these variables were not captured in our retrospective records and could not be adjusted for, so residual confounding from this source cannot be excluded and is a priority for prospective study in the diabetes-enriched Gulf region (17, 40). The likely direction of this bias is toward attenuation: the high background prevalence of diabetes in Gulf populations may raise baseline MDW in the other-infection comparator group, narrowing the separation between groups and further compressing MDW’s apparent discrimination Sixth, mortality data were not available; therefore, we could not assess the prognostic value of MDW, despite signals in the wider literature (38, 41). Finally, MDW is currently restricted to Beckman Coulter DxH-series analyzers, which limits its direct portability to other platforms (17, 42).

## Conclusion

This first UAE evaluation of MDW supports its position as a low-cost, rapidly available sepsis screening biomarker generated automatically from routine CBC. MDW on its own provided moderate discrimination in an infection-enriched cohort; the reported AUCs therefore reflect this design and are expected to be lower than those obtained in unselected emergency-department populations. Combined with qSOFA at the bedside, it produced the strongest performance in our analysis, which was statistically equivalent to a CRP-based composite. The high sensitivity at the ≥ 20 cutoff supports the established rule-out role of the MDW. In multivariable analysis, MDW remained statistically independent of CRP and PCT, with no evidence of collinearity (all VIF < 1.8); we interpret this as statistical independence within this model rather than proof of biological non-redundancy, since attenuation of CRP and PCT may also reflect the modest sample size. Prospective multicenter studies from the Gulf region are needed to derive region-specific cutoffs and test the clinical impact of MDW-guided sepsis pathways on patient outcomes.

## Data Availability

The raw data supporting the conclusions of this article will be made available by the authors, without undue reservation.

## Supplementary material

SUPPLEMENTARY FIGURE S1 Spearman rank correlation heatmap among biomarkers and clinical scores. Pairwise Spearman correlation coefficients (ρ) are shown for MDW, CRP, PCT, WBC, qSOFA, SIRS, and age. MDW correlates moderately with PCT (*ρ* = 0.62) and CRP (ρ = 0.41) but only weakly with qSOFA (ρ = 0.07) and SIRS (ρ = 0.01), supporting the interpretation that MDW carries inflammatory information distinct from clinical severity scores.

SUPPLEMENTARY FIGURE S2 Calibration plot for the full multivariable logistic-regression model. Observed proportions with sepsis are plotted against predicted probabilities across deciles of predicted risk; the dashed line denotes perfect calibration. The model showed good agreement between predicted and observed risk (Hosmer–Lemeshow χ² = 10.28, df = 8, *p* = 0.25; bootstrap calibration slope 0.96).
