# Supplementary material for: Diagnostic accuracy and severity prediction of monocyte distribution width in sepsis: a retrospective study from the United Arab Emirates
Source: Front Med (Lausanne). 2026 Aug 5;13:1891671. doi: 10.3389/fmed.2026.1891671 (PMC13486234; doi:10.3389/fmed.2026.1891671)
Supplement: Supplementary file 1 [file Data_Sheet_1.PDF]

STARD 2015 Checklist

Diagnostic Accuracy and Severity Prediction of Monocyte Distribution Width in Sepsis: A Retrospective Study from the United Arab Emirates

Reporting checklist for diagnostic accuracy studies (STARD 2015). Section/paragraph references point to locations in the revised manuscript. [Authors: confirm page numbers after final typesetting.]

#	Item	Location in manuscript
1	Identification as a study of diagnostic accuracy using at least one measure of accuracy (e.g. sensitivity, specificity, AUC)	Title; Abstract
2	Structured summary of design, methods, results, and conclusions	Abstract
3	Scientific and clinical background, including the intended use and clinical role of the index test	Introduction, ¶1–5
4	Study objectives and hypotheses	Introduction, final ¶
5	Whether data collection was planned before the index test and reference standard were performed (prospective) or after (retrospective)	Methods – Study design (retrospective)
6	Eligibility criteria	Methods – Population; Figure 1
7	On what basis potentially eligible participants were identified (e.g. symptoms, results from previous tests, inclusion in registry)	Methods – Population (ICD-10 R65/R65.21 screening of consecutive admissions)
8	Where and when potentially eligible participants were identified (setting, location, dates)	Methods – Study design (Rashid Hospital; study period)
9	Whether participants formed a consecutive, random, or convenience series	Methods – Population (consecutive)
10a	Index test, in sufficient detail to allow replication	Methods – Data and definitions (MDW, UniCel DxH 900)
10b	Reference standard, in sufficient detail to allow replication	Methods – Data and definitions (Sepsis-3 criteria)
11	Rationale for choosing the reference standard	Methods – Data and definitions; Introduction
12a	Definition of and rationale for test positivity cut-offs or result categories of the index test, distinguishing pre-specified from exploratory	Methods – Statistical analysis; Results (Youden + FDA-cleared ≥20)
12b	Definition of and rationale for test positivity cut-offs or result categories of the reference standard	Methods – Data and definitions (Sepsis-3)
13a	Whether clinical information and reference standard results were available to the performers/readers of the index test	Methods – Population (MDW not used clinically; diagnosis made without reference to MDW)
13b	Whether clinical information and index test results were available to the assessors of the reference standard	Methods – Population (treating-clinician diagnosis independent of MDW)
14	Methods for estimating or comparing measures of diagnostic accuracy	Methods – Statistical analysis (ROC, DeLong, logistic regression)
15	How indeterminate index test or reference standard results were handled	Methods – Data and definitions (missing data; blood-culture classification); Supplementary sensitivity analysis

16	How missing data on the index test and reference standard were handled	Methods – Statistical analysis; Supplementary Table S2
17	Any analyses of variability in diagnostic accuracy, distinguishing pre-specified from exploratory	Results – Subgroup analyses (framed as exploratory); Figure 3
18	Intended sample size and how it was determined	Methods – Population (Hanley–McNeil power calculation)
19	Flow of participants, using a diagram	Figure 1 (patient flow)
20	Baseline demographic and clinical characteristics of participants	Results – Patient characteristics; Table 1
21a	Distribution of severity of disease in those with the target condition	Results; Table 1; Figure 1
21b	Distribution of alternative diagnoses in those without the target condition	Results; Supplementary Table S1 (sources of infection)
22	Time interval and any clinical interventions between index test and reference standard	Methods – Data and definitions (admission laboratory values)
23	Cross tabulation of the index test results by the results of the reference standard	Table 2; Results – Diagnostic performance
24	Estimates of diagnostic accuracy and their precision (e.g. 95% CI)	Table 2; Results (AUC with 95% CI)
25	Any adverse events from performing the index test or reference standard	Not applicable (non-interventional CBC parameter)
26	Study limitations, including sources of potential bias, statistical uncertainty, and generalisability	Discussion – Limitations
27	Implications for practice, including the intended use and clinical role of the index test	Discussion; Conclusion
28	Registration number and name of registry	[Authors to confirm — institutional approval cited; trial registry N/A for retrospective]
29	Where the full study protocol can be accessed	[Available from corresponding author]
30	Sources of funding and other support; role of funders	Declarations – Funding
