# Supplementary material for: Diagnostic accuracy and severity prediction of monocyte distribution width in sepsis: a retrospective study from the United Arab Emirates
Source: Front Med (Lausanne). 2026 Aug 5;13:1891671. doi: 10.3389/fmed.2026.1891671 (PMC13486234; doi:10.3389/fmed.2026.1891671)
Supplement: Supplementary file 2 [file Table_1.DOCX]

**Supplementary Table S1.** Extended baseline characteristics; SIRS scores and consolidated sources of infection, stratified by diagnosis group.

| **Variable** | **Sepsis (n=53)** | **Septic Shock (n=21)** | **Other (n=66)** | **p** |
| --- | --- | --- | --- | --- |
| SIRS score, median (IQR) | 1 (1–2) | 2 (1–3) | 1 (1–2) | 0.198 |
| *Source of infection, n (%)* |  |  |  |  |
| Respiratory | 26 (49.1) | 8 (38.1) | 35 (53.0) | — |
| Urinary tract | 15 (28.3) | 9 (42.9) | 1 (1.5) | — |
| Gastrointestinal | 4 (7.5) | 0 (0.0) | 19 (28.8) | — |
| Central nervous system | 1 (1.9) | 0 (0.0) | 5 (7.6) | — |
| Cardiac / endocarditis | 0 (0.0) | 0 (0.0) | 3 (4.5) | — |
| Skin / soft tissue | 1 (1.9) | 0 (0.0) | 2 (3.0) | — |
| Bloodstream (primary) | 3 (5.7) | 1 (4.8) | 0 (0.0) | — |
| Other / unspecified | 3 (5.7) | 3 (14.3) | 1 (1.5) | — |

*SIRS, Systemic Inflammatory Response Syndrome. SIRS scores are presented as median (interquartile range) and were compared between groups using the Kruskal–Wallis test. Sources of infection were derived from electronic medical record review and consolidated into the categories shown. Some patients had more than one documented site of infection; primary source as judged by the treating team was used. χ² testing of source-of-infection distribution across groups gave a global p < 0.001, driven principally by the predominance of urinary tract infection in sepsis and septic shock groups and gastrointestinal infection in the other-infection group.*
